# Vegetables and Fruit as a Reservoir of β-Lactam and Colistin-Resistant Gram-Negative Bacteria: A Review

**DOI:** 10.3390/microorganisms9122534

**Published:** 2021-12-08

**Authors:** Widad Chelaghma, Lotfi Loucif, Mourad Bendahou, Jean-Marc Rolain

**Affiliations:** 1 Laboratoire de Microbiologie Appliquée à l’Agroalimentaire au Biomédical et à l’Environnement, Département de Biologie, Faculté des Sciences de la Nature et de la Vie, des Sciences de la Terre et l’Univers, Université Abou Bekr Belkaid, Tlemcen 13000, Algeria; widadc2014@hotmail.com (W.C.); bendahou63@yahoo.fr (M.B.); 2 Laboratoire de Biotechnologie des Molécules Bioactives et de la Physiopathologie Cellulaire (LBMBPC), Faculté des Sciences de la Nature et de la Vie, Université de Batna 2, Batna 05000, Algeria; 3 Microbes, Evolution, Phylogénie et Infection (MEPHI), Institut de Recherche pour le Développement (IRD), Faculté de Médecine et de Pharmacie, Aix-Marseille Université, 13000 Marseille, France; jean-marc.rolain@univ-amu.fr; 4 IHU Méditerranée Infection, Assistance Publique des Hôpitaux de Marseille, 13000 Marseille, France

**Keywords:** β-lactamases, mobile colistin resistance, Gram-negative bacteria, vegetables, fruit

## Abstract

Antibacterial resistance is one of the 2019 World Health Organization’s top ten threats to public health worldwide. Hence, the emergence of β-lactam and colistin resistance among Gram-negative bacteria has become a serious concern. The reservoirs for such bacteria are increasing not only in hospital settings but in several other sources, including vegetables and fruit. In recent years, fresh produce gained important attention due to its consumption in healthy diets combined with a low energy density. However, since fresh produce is often consumed raw, it may also be a source of foodborne disease and a reservoir for antibiotic resistant Gram-negative bacteria including those producing extended-spectrum β-lactamase, cephalosporinase and carbapenemase enzymes, as well as those harboring the plasmid-mediated colistin resistance (mcr) gene. This review aims to provide an overview of the currently available scientific literature on the presence of extended-spectrum β-lactamases, cephalosporinase, carbapenemase and mcr genes in Gram-negative bacteria in vegetables and fruit with a focus on the possible contamination pathways in fresh produce.

## 1. Introduction

Fresh produce is considered a good source of minerals, vitamins, phytonutrients and dietary fiber. Accordingly, there is a consensus that a diet rich in vegetables and fruit may decrease the risk of heart diseases and protect against some types of cancer [1]. In 2003, the Food and Agriculture Organization (FAO) of the United Nations and the World Health Organization (WHO) started an initiative worldwide to promote fruit and vegetable intake for health, with a recommended minimum consumption of 400 g of vegetables and fruit per day [2]. Following this recommendation, the intake of fresh produce as ingredients in healthy diets has been increasing and has gained popularity globally [3]. Consequently, the consumption of contaminated fresh produce, such as vegetables and fruit eaten raw, has been associated with an increasing number of outbreaks of foodborne disease [1]. In addition, fresh produce represents a route of human exposure to antibiotic-resistant bacteria and has often served as a reservoir of antibiotic resistance genes, representing a major public health threat [3,4].

In this context, one of the main public health preoccupations worldwide is the emergence of Gram-negative bacteria displaying resistance to oxyimino-cephalosporins (3GCs), carbapenem and colistin [3]. β-lactams, essentially extended-spectrum cephalosporins and carbapenems, are the main therapeutic choices to treat infections caused by resistant Gram-negative bacteria [5,6,7]. However, resistance to these antibiotic drugs has been increasing in recent years mostly through β-lactamase production. Various β-lactamases have been identified worldwide, including penicillinases, extended-spectrum β-lactamases (ESBLs), cephalosporinases (AmpC), and carbapenemases [7]. Given these circumstances, the approved alternative is colistin, but its re-use in clinical practice has led to the appearance of colistin-resistant bacteria, particularly through horizontal transfer (mcr) [8].

The transfer of these multidrug-resistant Gram-negative bacteria to fresh produce may occur during production via animal manure, through the use of contaminated irrigation water, or be linked to humans during the post-harvest stage, as well as during transport, conservation and processing by handlers [9]. The ingestion of antibiotic-resistant bacteria poses a potential public health concern since they are able to colonize the gut and exchange resistance genes with intestinal bacteria during their passage through the intestines which facilitates their further dissemination in the environment [3]. Extended-spectrum β-lactamase, cephalosporinase and carbapenemase producers as well as mcr gene-producing Gram-negative bacteria isolated from fresh vegetables and fruit have been reported in several countries around the world [3,4,10,11].

Thus, the aim of this review is to highlight the current situation of the worldwide dissemination of ESBL, cephalosporinase, carbapenemase and mcr gene-producing Gram-negative bacteria from fresh vegetables and fruit, their genetic characteristics, and possible contamination pathways.

## 2. β-Lactam Resistance and Gram-Negative Bacteria

β-lactam resistance in Gram-negative bacteria can be attributed to two main mechanisms, these include the acquisition of β-lactamase genes, as well as the modification of the target (penicillin-binding proteins) [12]. β-lactamase enzymes have played an important clinical role and have served as the principal resistance mechanism detected for β-lactam drugs [13,14].

The first enzyme detected presenting β-lactamase activity originated from *Bacillus coli* in 1940, currently supposed to be the class C, AmpC chromosomal cephalosporinase from *Escherichia coli* [14]. Given this, various extended-spectrum cephalosporins were introduced in the 1980s, which were stable against penicillinase hydrolysis, such as TEM-1 (TEMoniera) and SHV-1 (sulfhydryl variable). A few years later, Enterobacteriaceae species developed several derivatives of TEM-1, TEM-2 and SHV-1; these variants extended their hydrolysis spectrum to include oxyimino-cephalosporins, hence the term ‘extended-spectrum’ β-lactamases (ESBL) [14,15]. Afterwards, a novel variant of the ESBL family named Cefotaximase-Munchen (CTX-M) was described, which became the predominant ESBL in enterobacterial species worldwide [14], as well as the family of Guyana extended-spectrum β-lactamases (GES) reported as ESBL variants in 2000 [16]. The β-lactamases belonging to Ambler class C, called cephalosporinases, are derived from the ampC gene in the chromosome of various Enterobacteriaceae species [17,18]. In the early 1990s, plasmid-encoded AmpC cephalosporinases were described in species lacking an inducible AmpC enzyme. Afterwards, plasmid-mediated AmpC, such as Dharhan hospital (DHA), cephamycinase (CMY), cefoxitinase (FOX), moxalactamase (MOX) and Ambler Class C (ACC), were reported worldwide [19].

In this worrisome situation, carbapenems were introduced to clinics in the late 1980s and showed significant activity in the treatment of infections caused by AmpC and ESBL-producing Gram-negative bacteria [16,20]. The first carbapenemase reported in Enterobacteriaceae was the *Serratia marcescens* enzyme (SME-1) in London in 1982. Since then, various carbapenemase enzymes belonging to the Ambler class A β-lactamases have been reported, including imipenemase (IMI-1) and non-metallocarbapanemase class A (NmcA); however, the *K. pneumoniae*-carbapenemase (KPC) type was the most commonly found [5,16,21]. On the other hand, the first MBL variant was discovered in *Bacillus cereus* in 1966 and was called the BCII enzyme. Until 1989, only four MBL enzymes had been identified and were all chromosomally encoded, therefore they were deemed clinically negligible. Afterwards, various plasmid-encoding class B carbapenemases were described, such as Imipenem-resistant *Pseudomonas*-type carbapenemases (IMP),Verona integron-encoded MBL (VIM),and recently, New Delhi MBL (NDM) [22,23].In class D β-lactamases, several variants with relatively weak carbapenemase activity have also been reported as carbapenemase enzymes, including OXA-48, OXA-58, OXA-24/40 and OXA-23 [22]. In Enterobacteriaceae, class D carbapenemases are mainly represented by the OXA-48-like enzymes [24].

## 3. Colistin Resistance in Gram-Negative Bacteria

Colistin is a cationic polypeptide antibiotic belonging to the polymyxin family [25]. It was described initially in 1947 in *Paenibacillus polymyxa*, and it is commonly used in human and veterinary medicines, plant cultivation and animal husbandry [25,26,27]. Although in the 1970s its use was discontinued due to its neuro- and renal toxicity, it was reintroduced in the mid-2000s as a last line therapeutic option for the treatment of extensively drug-resistant (XDR) Gram-negative infections, such as those caused by carbapenem-resistant GNB [26,28].

The initial target site of colistin is lipopolysaccharide (LPS), more exactly lipid A, located in the outer membrane, which plays a major role in cell permeability. The electrostatic interaction between the cationic region of colistin, which is from the diamino-butyric acid (Dab) residues, and the negatively charged phosphate groups of lipid A, replace the magnesium and calcium ions previously united with the phosphate group. This destabilizes the lipid A and increases the permeability of the outer membrane, leading to the entry of colistin by a self-promoted uptake mechanism and eventual bacterial death [26,29]. Another antibacterial mechanism is the inhibition of a crucial respiratory enzyme, the type II NADH-quinone oxidoreductase (NDH-2) in the bacterial cell membrane [29]. The increased use of colistin has led to the emergence of colistin-resistant strains worldwide [25]. Colistin resistance is mainly achieved by modification of LPS, and consequently the reduced or absent affinity for colistin. This mechanism, although universal in Gram-negative bacteria, may differ between species. The lipid A of LPS undergoes changes, essentially due to the addition of positively charged residues such as phosphoethanolamine (PEtn) and/or 4-amino-4-deoxy-L-arabinose (L-Ara4N). These molecules decrease the overall negative charge of LPS, leading to a smaller electrostatic interaction with the positive charges of colistin that inhibits cell lysis [26,30].

Previously, the genes responsible for most of these additions were thought to be due to chromosomal mutations in genes of a two-component regulatory system, such as pmrAB, PhoPQ, and mgrB, which are not transferable [30]. In late 2015, Liu et al. described the mobilized colistin resistance (mcr-1) gene in an *E. coli* isolate recovered from livestock in China [8,31]. MCR-1 confers resistance by modifying the colistin target through the action of phosphoethanolamine transferase, which ensures the transfer of phosphoethanolamine (PEA) onto the glucosamine saccharide of lipid A, contributing as in chromosomal resistance to reduce the net negative charge of lipid A and consequently, colistin binding [32]. After the discovery of the mcr-1 gene, nine other mcr gene types (mcr-2 to mcr-10) were identified. The second mobile colistin resistance gene, mcr-2, was found initially in *E. coli* strains isolated from pigs and calves in Belgium [33]. The gene mcr-3 was identified in *E. coli* from pigs in China [34], and mcr-4 was reported in *Salmonella enterica* serovar *Typhimurium* strains isolated from pigs in Italy [35]. In 2017, anovel transposon-associated phosphoethanolamine transferase gene (mcr-5) was described in d-tartrate-fermenting *Salmonella enterica* subsp. *enterica* serovar *paratyphi B* in Germany [36]. In 2018, further variants were described; mcr-6 was identified in *Moraxella* spp. isolated from pigs in Great Britain [37], while mcr-7 and mcr-8 were described in *K. pneumoniae* strains isolated from animals (chickens and pigs) in China [38,39]. In 2019, a novel variant mcr-9 was reported in a *Salmonella enterica* serovar *Typhimurium* strain isolated from a human in Washington State in 2010 [40] and more recently, Wang et al. reported the detection of an mcr-10 variant in an *Enterobacter roggenkampii* clinical strain in China [41].

## 4. Literature Search Strategy and Data Collection

The dissemination of extended-spectrum β-lactamase, cephalosporinase, carbapenemase and MCR-producing Gram-negative bacteria in fresh produce is a major public health threat, since they are a very suitable pathway for the spread of antibiotic-resistant bacteria from farm to fork. Until June 2021, thirty-three molecular studies have revealed the isolation of Gram-negative bacteria producing β-lactamase and mcr genes on fresh vegetables and fruit. They have been used and are accessible through the PubMed database using the following keywords: ‘‘ESBL’’, ‘‘AmpC’’, ‘‘KPC’’, ‘‘VIM’’, ‘‘NDM’’, ‘‘IMP’’, ‘‘OXA-48”, ‘‘mcr’’, ‘‘carbapenem resistance’’, ‘‘fresh vegetables’’, ‘‘vegetables’’ and ‘‘fruit’’.

## 5. Vegetable and Fruit Isolates with ESBL and Cephalosporinase Genes

**Figure 1:**
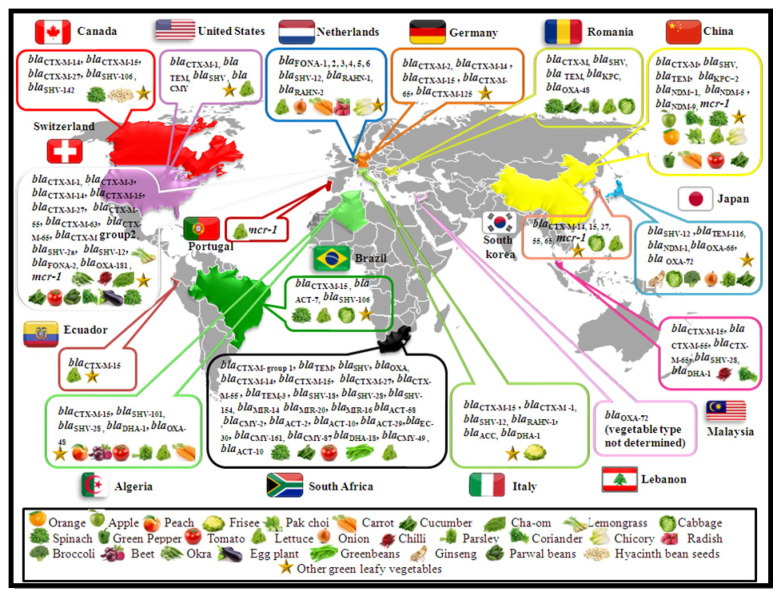
Worldwide distribution of extended-spectrum β-lactamase, cephalosporinase, carbapenemase and mcr-producing GNB on fresh vegetables and fruit.

A total of nineteen molecular studies reporting the isolation of ESBL-producing Gram-negative bacteria and AmpC genes from vegetables and fruit have been described (Figure 1, Table 1). The first report of ESBL-producing GNB isolates from vegetables and fruit was reported in 2014 in The Netherlands. These bacteria were reported on six vegetable types that are consumed raw (bunched carrots, blanched celery, endive, chicory, iceberg lettuce and radish), and from iceberg lettuce farms. In that study, the *bla*_FONA-5_ gene was detected among *Serratia fonticola* isolates on iceberg lettuce from a farm. In addition, 35 *Rahnella aquatilis* strains harboring the *bla*_RAHN_ gene were identified. Of the 35 isolates, 34 strains were producing the *bla*_RAHN-1_, and only one *R. aquatilis* strain carried the *bla*_RAHN-2_ gene [42]. After this publication, this level of resistance has been reported in Europe, Africa, Asia and America. Like isolates from humans, animals and the environment, the CTX-M family is the most prevalent type of ESBL-producing Enterobacteriaceae found in vegetables. Similarly, in an Italian study carried out on fresh vegetables, the authors refer to the detection of different ESBL enzymes, including CTX-M-15, CTX-M-1, SHV-12 and RAHN-1 in twenty isolates (the *bla*_CTX-M-15_ gene in *C. freundii*, *E. coli* and *Pantoea agglomerans*, the *bla*_CTX-M-1_ gene in *Enterobacter cloacae*, the *bla*_SHV-12_ in *E. coli* and *bla*_RAHN-1_ in *R. aquatilis*). Whereas only four isolates displayed AmpC production, among the four strains obtained, two *Hafnia alvei* isolates carried a *bla*_ACC_ gene and two *E. cloacae* harbored a *bla*_DHA-1_ gene [43]. A study in The Netherlands investigated the prevalence of third-generation cephalosporin (3GC) resistant Gram-negative bacteria on fresh vegetables. A total of 27 *Serratia* spp. isolates with an ESBL phenotype harboring a *bla*_FONA_ variant were obtained, including *bla*_FONA-1_ (18.5%), *bla*_FONA-2_ (37.0%), *bla*_FONA-3_ (7.4%), *bla*_FONA-4_ (7.4%), *bla*_FONA-5_ (18.5%) and *bla*_FONA-6_ (11.1%). The *bla*_SHV-12_ gene was detected in one *E. coli* and two *Enterobacter* spp. strains; however, one *R. aquatilis* strain harbored the *bla*_RAHN-1_ gene [3]. In Switzerland, two studies reported the detection of *bla*_ESBL_ genes on vegetable samples. In the first study, the authors evaluated the presence of ESBL-producing Enterobacteriaceae in 68 vegetables imported from the Dominican Republic, India, Thailand and Vietnam via the national airport in Zürich, and 101 samples were purchased in the city of Zürich. In total, 60 ESBL producers were retrieved, including *bla*_CTX-M_- and *bla*_SHV_-producing *E. coli* (*bla*_CTX-M-15_, *bla*_CTX-M-55_, *bla*_CTX-M-14_, *bla*_CTX-M-65_, *bla*_CTX-M-1_ and *bla*_SHV-12_) and *K. pneumoniae* strains (*bla*_CTX-M-15_, *bla*_CTX-M-14_, *bla*_CTX-M-3_, *bla*_CTX-M-27_, *bla*_CTX-M-63_, *bla*_SHV-2_, *bla*_SHV-2a_, and *bla*_SHV-12_). Moreover, *bla*_CTX-M-15_ and *bla*_SHV-2_ genes were identified in *E. cloacae*, *E. aerogenes* and *C. sakazakii,* respectively [10]. The second study reported the detection of CTX-M group 2, CTX-M-15 and FONA-2 in *Kluyvera ascorbata*, *E. cloacae* and *S. fonticola* isolates from diced tomato, chopped chives and spinach, respectively [44]. A study from Germany described the isolation of seven ESBL-producing *E. coli* isolates collected by food safety inspectors during 2011–2013 from markets, producers and supermarkets. Of the seven isolates, two strains were positive for *bla*_CTX-M-14_ and two other isolates harbored *bla*_CTX-M-15_ genes. However, three remaining strains were positive for *bla*_CTX-M-65_, *bla*_CTX-M-125_ and *bla*_CTX-M-2_ genes, respectively [45]. In addition, the *bla*_TEM_, *bla*_SHV_, *bla*_CTX-M_ and *bla*_DHA_ genes were also reported in Romania in different Enterobacteriaceae species (*S. marcescens*, *E. cloacae*, *E. coli*, *Klebsiella oxytoca* and *Proteus vulgaris*) [46].

In Africa, the first recorded ESBL and/or cephalosporinase-positive GNB was observed in 2019 in South Africa. In this report, 545 vegetable samples including spinach, cucumbers, tomatoes, green beans and lettuce, were collected from street-trading greengrocers, mobile trolley vendors, formal retailers and vendors at two farmers markets from September 2017 to May 2018. ESBL genes were detected in 39 strains, while AmpC production was observed in 20 strains belonging to 10 genera of Enterobacteriaceae including *S. fonticola*, *Serratia marcescens*, *E. coli*, *E. cloacae*, *Enterobacter asburiae*, *Enterobacter cowanii*, *Enterobacter ludwigii*, *R. aquatilis*, *K. pneumoniae*, *Klebsiella oxytoca*, *Citrobacter freundii*, *Proteus mirabilis* and *Proteus penneri*. Different *bla*_CTX-M_ genes were obtained, including *bla*_CTX-M-14_ (*n* = 15), *bla*_CTX-M-15_ (*n* = 6), *bla*_CTX-M-27_ (*n* = 4) and *bla*_CTX-M-55_ (*n* = 3). In addition, the *bla*_TEM-3_ gene (*n* = 3), as well as *bla*_SHV_ genes encoding *bla*_SHV-18_ (*n* = 6), *bla*_SHV-28_ (*n* = 1), and *bla*_SHV-154_ (*n* = 1) were detected. Three isolates carried more than one ESBL gene; two strains (*E. cowanii* and *E. coli*) harbored the *bla*_TEM-3_ gene in association with *bla*_SHV-18_ and *bla*_CTX-M-14_ genes, respectively, while one *E. coli* isolate carried *bla*_CTX-M-14_, *bla*_SHV-18_ and *bla*_TEM-3_ genes. AmpC genetic determinants were observed in 18 of 58 (31%) isolates, 17 strains carried only one pAmpC gene, including *bla*_MIR-20_ (*n* = 4), *bla*_MIR-16_ (*n* = 3), and *bla*_ACT-58_ (*n* = 2), and one isolate each harbored *bla*_MIR-14_, *bla*_CMY-2_, *bla*_ACT-2_, *bla*_ACT-10_, *bla*_ACT-29_, *bla*_EC-30_, *bla*_CMY-161_ or *bla*_CMY-87_, respectively. As well, one *P. penneri* isolate harbored three AmpC genetic determinants (*bla*_DHA-18_, *bla*_CMY-49_ and *bla*_ACT-10_). Among these 17 isolates, five strains (*Enterobacter* spp. (*n* = 2), *R. aquatilis* (*n* = 1), *E. coli* (*n* = 1) and *S. fonticola* (*n* = 1)) also carried ESBL genes [9]. Another report from South Africa described the detection of twenty enterobacterial isolates, identified as *E. asburiae*, *E. coli*, *K. pneumoniae*, *R. aquatliis* and *S. fonticola,* harboring different ESBL and AmpC genes, including *bla*_CTX-M-group1_, *bla*_TEM_, *bla*_SHV_, *bla*_OXA_ and *bla*_CIT_ genes [47]. In Algeria, Mesbah Zekar et al. reported the identification of multi-drug resistant *K. pneumoniae* isolates in fresh fruit and vegetables purchased in Bejaia city. In this study, eleven *K. pneumoniae* isolates harbored multiple ESBL genes, and *bla*_CTX-M-15_, *bla*_OXA-1_, *bla*_SHV-101_ and *bla*_SHV-28_ were described. In addition, two *K. pneumoniae* strains coharbored *bla*_DHA-1_ with ESBL genes [48].

In Asia, Usui et al. analyzed 130 samples of fresh vegetables collected from seven supermarkets in Japan, 10 out of the 130 samples contained ESBL-producing *Pseudomonas* spp. including; *P. hunanensis*, *P. putida*, *P. parafulva*, *P. beteli*, *P. mosselii*, *P. paralactis* and *P. arsenicoxydans*. These isolates harbored the *bla*_SHV-12_ or *bla*_TEM-116_ ESBL gene [49]. In China, a nationwide survey investigated the prevalence of ESBL-producing Enterobacteriaceae from retail food, where four isolates were obtained. Three were identified as *E. coli* and one as *C. freundii* isolated from retail vegetables, including tomatoes, cucumber and coriander. The *C. freundii* isolate carried *bla*_CTX-M_ and *bla*_OXA_ genes, while two *E. coli* isolates harbored *bla*_CTX-M_ and *bla*_SHV_ genes and one other *E. coli* strain carried *bla*_CTX-M_, *bla*_SHV_ and *bla*_TEM_ genes [7]. In Malaysia, ESBL or AmpC genes were detected in two *E. coli* (*bla*_CTX-M-55_ and *bla*_CTX-M-65_) and two *K. pneumoniae* isolates (*bla*_CTX-M-15_, *bla*_SHV-28_ and *bla*_DHA-1_) from coriander and chili pepper respectively [50]. In addition, different CTX-M variants were described from *E. coli* isolates in South Korea including CTX-M-14, CTX-M-15, CTX-M-27, CTX-M-55 and the CTX-M-65 variant [51].

On the American continent, different Enterobacteriaceae isolates harbored *bla*_SHV_, *bla*_TEM_ and *bla*_CTX-M-1_ as well as *bla*_CTX-M_ and *bla*_CMY_ genes and were detected from iceberg lettuce and leafy greens, respectively in the United States [52,53]. Moreover, seven *E. coli* isolates carrying the *bla*_CTX-M-15_ gene were reported from leaf lettuce, alfalfa and parsley/cilantro in Ecuador [54], while *bla*_CTX-M-14_, *bla*_CTX-M-15_, *bla*_CTX-M-27_, *bla*_SHV-106_ and *bla*_SHV-142_-positive Enterobacteriaceae were reported in Canada from imported vegetable samples [55], and the *bla*_CTX-M-15_ gene in Brazil [56].

**Table 1:** ESBL and cephalosporinase genes reported in Gram-negative bacteria isolates from vegetables and fruit worldwide.

Vegetable Type	ESBL/AmpC Gene	Isolation Period	Species	Isolates Number	Country	Other Antibiotic Resistance Genes	Sequence Type	References
Lettuce	*bla*_FONA-5_	2011	*Serratia fonticola*	1	The Netherlands	ND	ND	[42]
	*bla*_RAHN-2_		*Rahnella aquatilis*	1				
	*bla*_CTX-M-15_	2013–2014	*Klebsiella pneumoniae*	1	Algeria	aph(3′)-Ia, aadA2, strB, strA, qnrS1, oqxB, oqxA, fosA, mph(A), catA2, sul1, sul2, tet(A), dfrA12	ST219	[48]
	*bla*_DHA-1_ *bla*_SHV-101_		*K. pneumoniae*	1		*bla*_OXA-1_, aac(6′)Ib-cr, aph(3′)-Ia, aac(6′)Ib-cr, qnrB4, oqxB, oqxA, fosA, mph(A) catB3, ARR-3, sul1	ST882	
	*bla*_SHV-28_, *bla*_CTX-M-15_,		*K. pneumoniae*	1		*bla*_OXA-1_, aac(6′)Ib-cr, aac(3)-Iia, aac(6′)Ib-cr, qnrB66, oqxB, oqxA, fosA, catB3, dfrA14.	ST14	
	*bla*_CTX-M-15_	2015	*Escherichia coli*	1	Ecuador	dfrA1, aadA5	ST44	[54]
	*bla*_CTX-M-15_			1		None	ST44	
	*bla*_CTX-M-14_	2017–2018	*S. fonticola*	1	South Africa	ND	ND	[9]
	*bla*_SHV-154_		*S. marcescens*	1		ND	ND	
	*bla*_CTX-M-15_	2018	*E. coli*	1	South Korea	ND	ST2509	[51]
	*bla*_SHV_, *bla_TEM_*	2019	*Proteus vulgaris*	1	Romania	ND	ND	[46]
	*bla*_CTX-M-15_	ND	*K. pneumoniae*	1	Brazil	*bla*_OXA-1_, *bla*_SHV-110_, aac(3)IIa, aac(6′)-Ib-cr, opxAB, drfA14, catA1, tet(A), fosA, opxB	ST198	[56]
Butterhead lettuce	*bla*_FONA-1_ (1–6)	2012–2013	*S. fonticola*	ND	The Netherlands	ND	ND	[3]
Iceberg lettuce	*bla*_RAHN-1_	2011	*R. aquatilis*	ND	The Netherlands	ND		[42]
	*bla*_SHV_, *bla*_TEM_	2011–2012	*K. pneumoniae*	2	United States	ND	ND	[52]
	*bla*_CTX-M-1_		*S. marcescens*	1		ND	ND	
	*bla*_FONA-1_ (1–6)	2012–2013	*S. fonticola*	ND	The Netherlands	ND	ND	[3]
Tomato	*bla*_CTX-M_, *bla*_SHV_, *bla*_TEM_	2011–2014	*E. coli*	1	China	ND	ND	[7]
	*bla*_SHV-28_, *bla*_CTX-M-15_	2013–2014	*K. pneumoniae*	1	Algeria	aac(3)-Iia, qnrB66, oqxB, oqxA, fosA	ST14	[48]
	*bla*_SHV-28_, *bla*_CTX-M-15_,			1		*bla*_OXA-1_, aac(6′)Ib-cr, aac(3)-Iia, aac(6′)Ib-cr, qnrB66, oqxB, oqxA, fosA, catB3, dfrA14	ST14	
	*bla*_CMY-2_	2017–2018	*Citrobacter freundii*	1	South Africa	ND	ND	[9]
	*bla*_CTX-M-14_			1		ND	ND	
	*bla*_CTX-M-55_		*E. coli*	1		ND	ND	
	*bla*_CTX-M-14_			1		ND	ND	
	*bla*_CTX-M-14_			1		*bla*_SHV-1_, *bla*_TEM-215_	ND	
	*bla*_SHV-18_		*E. asburiae*	1		ND	ND	
	*bla*_MIR-14_			1		*bla*_SHV-26_	ND	
	*bla*_ACT-29_			1		ND	ND	
	*bla*_CTX-M-27_, *bla*_CTX-M-15_		*E. cloacae*	1		*bla*_SHV-26_	ND	
	*bla*_MIR-20_			1		ND	ND	
	*bla*_TEM-3_, *bla*_ACT-2_, *bla*_SHV-18_			1		*bla*_TEM-1_, *bla*_SHV-11_	ND	
	*bla*_SHV-18_, *bla*_TEM-3_		*E. cowanii*	1		ND	ND	
	*bla*_CTX-M-15_		*K. pneumoniae*	1		ND	ND	
	*bla*_ACT-10_		*K. oxytoca*	1		ND	ND	
	*bla*_CTX-M-55_		*Proteus mirabilis*	1		*bla*_TEM-215_	ND	
	*bla*_ACT-10_, *bla*_DHA-18_, *bla*_CMY-49_		*Pseudomonas penneri*	1		ND	ND	
	*bla*_SHV-18_		*R. aquatilis*	1		*bla*_TEM-215_	ND	
	*bla*_MIR-16_		*R. aquatilis*	1		ND	ND	
	*bla*_SHV-18_, *bla*_MIR-16_	2017–2018	*E. asburiae*	1	South Africa	*bla*_TEM-1_, *bla*_OXA-1_	ND	[9]
Diced tomato	*bla*_CTX-MGroup2_	2014	*Kluyvera ascorbata*	1	Switzerland	ND	ND	[44]
Spinach	*bla*_FONA-2_	2014	*S. fonticola*	1	Switzerland	ND	ND	[44]
	*bla*_CTX-M-group1_, *bla*_TEM_	2017	*E. asburiae*	1	South Africa	ND	ND	[47]
	*bla*_CTX-M-group1_, *bla*_TEM_, *bla*_SHV_, *bla*_OXA_		*E. coli*	2		ND	ND	
	*bla*_CTX-M-group1_, *bla*_TEM_, *bla*_SHV_, *bla*_OXA_		*K. pneumoniae*	3		ND	ND	
	*bla*_CTX-M-group1_, *bla*_TEM_		*E. asburiae*	1		ND	ND	
	*bla*_CTX-M-group1_, *bla*_TEM_, *bla*_SHV_, *bla*_OXA_		*E. coli*	2		ND	ND	
	*bla*_CTX-M-group1_, *bla*_TEM_, *bla*_SHV_, *bla*_OXA_		*K. pneumoniae*	3		ND	ND	
	*bla*_CTX-M-group1_		*R. aquatilis*	1		ND	ND	
	*bla*_CTX-M-group1_, *bla*_TEM_, *bla*_SHV_, *bla*_OXA_		*R. aquatilis*	1		ND	ND	
	*bla*_CTX-M-group1_, *bla*_TEM_, *bla*_SHV_		*R. aquatilis*	2		ND	ND	
	*bla*_CIT_		*S. fonticola*	3		ND	ND	
	*bla*_TEM_, *bla*_SHV_			1		ND	ND	
	*bla*_CTX-M-group1_, *bla*_TEM_, *bla*_SHV_, *bla*_OXA_			2		ND	ND	
	*bla*_CTX-M-group1_, *bla*_TEM_, *bla*_SHV_, *bla*_OXA_, *bla*_CIT_			1		ND	ND	
	*bla*_CTX-M-group1_, *bla*_SHV_			1		ND	ND	
	*bla*_CTX-M-group1_, *bla*_TEM_, *bla*_SHV_, *bla*_CIT_			2		ND	ND	
	*bla*_CTX-M-27_	2017–2018	*E. coli*	2	South Africa	ND	ND	[9]
	*bla*_MIR-20_			1		ND	ND	
	*bla*_SHV-18_, *bla*_CTX-M-15_, *bla*_TEM-3_			1		ND	ND	
	*bla*_CTX-M-14_, *bla*_TEM-3_			1		ND	ND	
	*bla*_CTX-M-14_			1		ND	ND	
	*bla*_CTX-M-15_			1		ND		
	*bla*_CTX-M-55_			1		ND	ND	
	*bla*_CTX-M-14_, *bla*_ACT-58_			1		ND	ND	
	*bla*_CTX-M-14_			2		ND	ND	
	*bla*_CTX-M-14_			2		*bla*_TEM-215_	ND	
	*bla*_ACT-58_		*E. asburiae*	1		*bla*_TEM-215_	ND	
	*bla*_CMY-87_		*E. ludwigii*	1		ND	ND	
	*bla*_CTX-M-27_, *bla*_EC-30_		*R. aquatilis*	1		ND	ND	
	*bla*_CTX-M-15_			1		*bla*_SHV-11_	ND	
	*bla*_CTX-M-15_, *bla*_SHV-28_		*S. fonticola*	1		ND	ND	
	*bla*_CTX-M-14_, *bla*_SHV-28_			1		ND	ND	
	*bla*_MIR-16_			1		*bla*_TEM-1_, *bla*_OXA-1_	ND	
	*bla*_CTXM-15_			1		*bla*_TEM-215_	ND	
	*bla*_SHV_, *bla_TEM_*	2019	*S. marcescens*	1	Romania	ND	ND	[46]
	*bla*_CTXM_		*E. cloacae*	1		ND	ND	
	*bla*_CTXM-15_	ND	*E. cloacae*	1	Brazil	*bla*_OXA-1_, *bla*_TEM-1B_, *bla*_ACT-7_, aac(3)-IIa, aac(6′)Ib-cr, ant(3′’)Ia, strA, strB, qnrB, sul2, tet(A), fosA.	ST927	[56]
	*bla*_CTXM-15_	ND	*E. coli*	1		*bla*_TEM-1B_, aac(3)IId, aadA5, strA, strB, tet(A)	ST14012	
Chopped Spinach	*bla*_CTXM14_, *bla*_SHV-142_	2017	*K. pneumoniae*	1	Canada	ND	ST261	[55]
	*bla*_CTXM-27_		*E. cloacae*	1		qnrB2, qnrS1, aac(6′)Ib cr	ND	
	*bla*_CTXM-27_		*E. aerogenes*	1		aac(6′) Ib cr	ND	
Ceylon spinach	*bla*_CTXM-14_	2014	*K. pneumoniae*	1	Switzerland	ND	ST37	[10]
Water spinach	*bla*_CTXM-15_		*K. pneumoniae*	1		ND	ST16	
Cucumber	*bla*_CTX-M_, *bla*_SHV_	2011–2014	*E. coli*	2	China	ND	ND	[7]
	*bla*_CTXM-15_	2014	*E. cloacae*	1	Switzerland	ND	ND	[10]
	*bla*_CTXM-15_		*E. coli*	1		ND	ST410	
	*bla*_TEM-116_	2015–2016	*P. mosselii*	1	Japan	ND	ND	[49]
	*bla*_MIR-20_	2017–2018	*E. cloacae*	1	South Africa	ND	ND	[9]
	*bla*_SHV-18_		*R. aquatilis*	1		*bla*_OXA-1_	ND	
	*bla*_CTXM_, *bla*_TEM_	2019	*E. coli*	1	Romania	ND	ND	[46]
	*bla_DHA_*		*E. cloacae*	1		ND		
Bitter cucumber	*bla*_CTXM-15_	2014	*E. coli*	1	Switzerland	ND	ST131	[10]
Coriander	*bla*_CTXM-55_	2011–2014	*E. coli*	2	China	ND	ST48, ST4680	[7]
	*bla*_CTX-M_, *bla*_OXA_		*Citrobacter freundii*	1		ND	ND	
	*bla*_CTX-M-55_	2018	*E. coli*	1	Malaysia	*bla*_TEM-1B_, aph(3 0)-Ia, aph(300)-Ib, aph(6)-Id, mdf(A), floR, ARR-2, sul2, tet(A), dfrA14	ST155	[50]
	*bla*_CTX-M-65_		*E. coli*	1		aac(3)-IV, aadA5, aph(4)-Ia, oqxA, oqxB, mdf(A), floR, sul1, sul2, tet(A), dfrA17	ST479	
Parsley	*bla*_SHV-28_, *bla*_CTX-M-15_, *bla*_OXA-1_	2013–2014	*K. pneumoniae*	1	Algeria	aac(6′)Ib-cr, aac(3)-Iia, aac(6′)Ib-cr, qnrB66, oqxB, oqxA, fosA, catB3, dfrA14	ST14	[48]
	*bla*_CTX-M-15_, *bla*_OXA-1_			1		aac(6′)Ib-cr, aac(3)-IIa, strB, strA, aac(6′)Ib-cr, oqxB, oqxA, fosA, catB3, sul2, tet(A), dfrA14	ST45	
	*bla*_SHV_	2019	*K. oxytoca*	1	Romania	ND	ND	[46]
Water parsley	*bla*_CTX-M-55_	2018	*E. coli*	1	South Korea	ND	ND	[51]
	*bla*_CTX-M-15_, *bla_TEM-1_*			1		ND	ST101	
	*bla*_CTX-M-14_, *bla_TEM-1_*			1		ND	ST354	
	*bla*_CTX-M-14_			1		ND	ST38	
Parsley/cilantro	*bla*_CTX-M-15_	2015	*E. coli*	1	Ecuador	None	ST410	[54]
				1		dfrA1, aadA5	ST44	
Soy sprouts	*bla*_CTX-M-65_	2011–2013	*E. coli*	1	Germany	floR, aac(6′)-Ib3, sul2, tet(A), fosA3	ST10	[45]
	*bla*_CTX-M-125_			1		aph(3′)-II, tet(A), fosA3	ST542	
	*bla*_CTX-M-14_			1		catA1, floR, aac(6′)Ib-cr, aph(3′)-Ia, aadA5, sul1, sul2, tet(A), dfrA17, fosA3	ST527	
	*bla*_CTXM-14_	2014	*K. pneumoniae*	1	Switzerland	ND	ST208	[10]
Sprouts-mixture	*bla*_CTX-M-15_	2011–2013	*E. coli*	1	Germany	*bla*_TEM-1_, qnrS1, strA, strB, sul2, tet(A), dfrA14	ST847	[45]
Alfalfa	*bla*_CTX-M-15_	2015	*E. coli*	1	Ecuador	dfrA1, aadA5	ST410	[54]
	*bla*_CTX-M-15_			1		None	ST44	
	*bla*_CTX-M-15_			1		None	ST44	
Alfalfa sprouts	*bla*_CTX-M-15_	2011–2013	*E. coli*	1	Germany	*bla*_TEM-1_, qnrS1, strA, strB, sul2, tet(A), dfrA14	ST410	[45]
Greenbeans	*bla*_CTX-M-14_	2017–2018	*E. coli*	2	South Africa	ND	ND	[9]
	*bla*_CTX-M-14_, *bla*_CMY-2_		*S. fonticola*	1		*bla*_TEM-215_	ND	
	*bla*_CTX-M-14_, *bla*_CMY-161_	2017–2018	*S. fonticola*	1		*bla*_TEM-215_	ND	
Curry leaves	*bla*_CTXM-15_	2014	*K. pneumoniae*	1	Switzerland	ND	ST307	[10]
	*bla*_CTXM-14_		*E. coli*	1		ND	ST38	
	*bla*_CTXM-15_		*K. pneumoniae*	1		ND	ST1742	
	*bla*_SHV-12_		*E. coli*	1		ND	ST1656	
	*bla*_CTXM-15_		*K. pneumoniae*	4		ND	ST1739, ST1741, ST1881, ST1740	
	*bla*_CTXM-1_		*E. coli*	1		ND	ST1555	
	*bla*_CTXM-15_			1		ND	ST4681, ST152	
	*bla*_CTXM-14_			1		ND	ST4679	
	*bla*_CTXM-55_			1		ND	ST10	
Mint	*bla*_CTX-M-15_, *bla*_SHV-28_	2013–2014	*K. pneumoniae*	1	Algeria	*bla*_OXA-1_, aac(6′)Ib-cr, aac(3)-Iia, aac(6′)Ib-cr, qnrB66, oqxB, oqxA, fosA, catB3, dfrA14	ST14	[48]
	*bla*_CTX-M-15_, *bla*_SHV-28_			1		*bla*_OXA-1_, aac(6′)Ib-cr, aac(3)-Iia, aac(6′)Ib-cr, qnrB66, oqxB, oqxA, fosA, catB3, dfrA14	ST14	
Chili	*bla*_CTXM-15_	2014	*E. coli*	1	Switzerland	ND	ST405	[10]
Green chili	*bla*_CTXM-15_		*E. cloacae*	1		ND	ND	[10]
	*bla*_CTXM-15_		*K. pneumoniae*	2		ND	ST1740, ST37	
	*bla*_CTXM-27_			1		ND	ST458	
Small chili	*bla*_CTXM-65_		*E. coli*	1		ND	ST167	
Chili pepper	*bla*_CTX-M-15_, *bla*_SHV-28_	2018	*K. pneumoniae*	1	Malaysia	*bla*_TEM-1B_, *bla*_OXA-1_, aac(3)-IIa, aac(6 0)-Ib-cr, aph(300)-Ib, aph(6)-Id, aac(6 0)-Ib-cr, oqxA, oqxB, qnrB1, fosA, catB3, sul2, tet(A), dfrA14	ST307	[50]
	*bla*_DHA-1_, *bla*_SHV-28_		*K. pneumoniae*	1		oqxA, oqxB, qnrS1, fosA, sul1, tet(A), dfrA1	ST101	
Hyacinth bean seeds	*bla*_CTXM-15_	2017	*E. coli*	1	Canada	ND	ST189	[55]
				1		*bla*_TEM-1_	ST226	
Ginseng	*bla*_TEM-116_	2015–2016	*Pseudomonas paralactis*	1	Japan	ND	ND	[49]
	*bla*_TEM-116_			1		ND	ND	
	*bla*_TEM-116_		*P. arsenicoxydans*	1		ND	ND	
Beets	*bla*_CTX-M-15_	2013–2014	*K. pneumoniae*	1	Algeria	aph(3′)-Ia, aadA2, strB, strA, nrS1, oqxB, oqxA, fosA, mph(A), catA2, sul1, sul2, tet(A), dfrA12	ST219	[48]
Carrot	*bla*_CTX-M-15_, *bla*_OXA-1_	2013–2014	*K. pneumoniae*	1		*bla*_TEM-1B_, aac(6′)Ib-cr, aac(3)-IIa, strB, strA, ac(6′)Ib-cr, qnrB66, oqxB, oqxA, fosA, catB3, sul2, dfrA14.	ST45	[48]
	*bla*_RAHN-1_	2011	*R. aquatilis*	ND	The Netherlands	ND	ND	[42]
Bunched carrot	*bla*_FONA_(1–6)	2012–2013	*S. fonticola*	ND	The Netherlands	ND	ND	[3]
Arugula	*bla*_RAHN-1_	2015–2016	*R. aquatilis*	4	Italy	ND	ND	[43]
	*bla*_CTX-M-15_		*C. freundii*	4		ND	ND	
	*bla*_ACC_		*Hafnia alvei*	2		ND	ND	
	*bla*_CTXM-15_ *bla*_SHV-106_	ND	*K. pneumoniae*	1	Brazil	*bla*_OXA-1_, *bla*_TEM-1B_, aac(6)Ib-cr, strA, strB, qnrB1, opxAB, gyrA, parC, tet(A), fosA.	ST2739	[56]
Egg plant	*bla*_CTXM-15_	2014	*K. pneumoniae*	1	Switzerland	ND	ST45	[10]
	*bla*_CTXM-15_			1		ND	ST307	
	*bla*_CTXM-15_		*E. cloacae*	1		ND	ND	
Chinese chive	*bla*_SHV-12_	2015–2016	*P. parafulva*	1	Japan	ND	ND	[49]
Chopped chives	*bla*_CTX-M-15_	2014	*E. cloacae*	1	Switzerland	ND	ND	[44]
Onion	*bla*_FONA-1_ (1–6)	2012–2013	*S. fonticola*	ND	The Netherlands	ND	ND	[3]
	*bla*_TEM-116_	2015–2016	*Pseudomonas beteli*	1	Japan	ND	ND	[49]
Broccoli	*bla*_TEM-116_	2015–2016	*P. hunanensis*	1	Japan	ND	ND	[49]
Cabbage	*bla*_TEM-116_	2015–2016	*P. hunanensis*	1	Japan	ND	ND	[49]
	*bla*_CTX-M_, *bla*_SHV_	2019	*E. cloacae*	1	Romania	ND	ND	[46]
	*bla*_CTXM-65_	2018	*E. coli*	1	South Korea	ND	2847	[51]
	*bla*_CTXM-15_	ND	*E. coli*	1	Brazil	*bla*_OXA-1_, *bla*_TEM-1B_, aac(3)IIa, aac(6′)Ib-cr, aadA5, gyrA, parC, sul1.	ST648	[56]
	*bla*_CTXM-15_	ND	*E. coli*	1		*bla*_OXA-1_, aac(6′)Ib-cr, aadA5, aac(6′)Ib-cr, sul1, drfA17, tet(B), mph(A)	ST38	
Cut cabbage	*bla*_SHV-12_	2015–2016	*P. hunanensis*	1	Japan	ND	ND	[49]
Bean sprout	*bla*_SHV-12_		*P. putida*	1		ND	ND	
Yard long beans	*bla*_CTXM-55_	2015–2016	*E. cloacae*	1	Japan	ND	ND	[49]
	*bla*_SHV-12_	2014	* Cronobacter sakazakii *	1	Switzerland	ND	ST3696	[10]
	*bla*_CTXM-14_		*E. coli*	1		ND	ND	
Holy Basil	*bla*_CTXM-15_	2014	*K. pneumoniae*	1	Switzerland	ND	ST36	
	*bla*_CTXM-65_		*E. coli*	1		ND	ST58	
Okra (marrow)	*bla*_CTXM-14_		*E. coli*	1		ND	ST38	
	*bla*_CTXM-15_		*E. coli*	2		ND	ST155, ST443	
Okra	*bla*_CTXM-15_		*K. pneumoniae*	2		ND	ST997, ST244	
	*bla*_CTXM-15_		*E. aerogenes*	1		ND	ND	
	*bla*_CTXM-15_		*E. cloacae*	2		ND	ND	
	*bla*_CTXM-15_		*E. coli*	2		ND	ST4682, ST4684	
Parwal beans	*bla*_CTXM-15_		*E. coli*	1		ND	ST641	
Peppermint	*bla*_CTXM-3_		*K. pneumoniae*	1		ND	ST15	
Cha-om (acacia)	*bla*_SHV-12_		*K. pneumoniae*	1		ND	ND	
	*bla*_CTXM-55_		*E. coli*	2		ND	ST167, ST393	
	*bla*_CTXM-14_		*E. coli*	1		ND	ST58	
Garlic chives	*bla*_CTXM-63_		*K. pneumoniae*	1		ND	ST1743	
	*bla*_CTXM-55_		*E. coli*	1		ND	ST226	
Lemongrass	*bla*_CTXM-14_		*K. pneumoniae*	1		ND	ST1530	
Sweet basil	*bla*_SHV-2a_		*K. pneumoniae*	1		ND	ST76	
Basil leaves	*bla*_CTXM-65_		*E. coli*	1		ND	ST4683	
Celery	*bla*_RAHN-1_	2011	*R. aquatilis*	34	The Netherlands	ND	ND	[42]
	*bla*_SHV-60_, *bla*_DHA-1_	2013–2014	*K. pneumoniae*	1	Algeria	*bla*_TEM-1D_, aadA1, strB, strA, qnrB4, oqxB, oqxA, fosA, sul1,tet(A), dfrA1	ST236	[48]
Lollo rosso leaves	*bla*_CTX-M-14_	2011–2013	*E. coli*	1	Germany	strA, strB, sul1, dfrA1	ST973	[45]
Lollo rosso and Lollo bionda leaves	*bla*_CTX-M-2_		*E. coli*	1		*bla*_TEM-1_, strA, strB, aadA5	ST120	
Blanched celery	*bla*_SHV-12_	2012–2013	*E. coli*	1	The Netherlands	ND	ND	[3]
	*bla*_FONA-1_		*S. fonticola*	ND		ND	ND	
Radish	*bla*_RAHN-1_	2012–2013	*R. aquatilis*	1	The Netherlands	ND	ND	[3]
	*bla*_FONA_(1–6)		*S. fonticola*	ND		ND	ND	
	*bla*_RAHN-1_	2011	*R. aquatilis*	ND		ND	ND	[42]
Chicory	*bla*_RAHN-1_	2011	*R. aquatilis*	ND	The Netherlands	ND	ND	[42]
Endive	*bla*_RAHN-1_	2011	*R. aquatilis*	ND		ND	ND	
	*bla*_FONA-1_ (1–6)	2012–2013	*S. fonticola*	ND	The Netherlands	ND	ND	[3]
Iceberg lettuce + arugula	*bla*_SHV-12_	2015–2016	*E. coli*	3	Italy	ND	ND	[43]
	*bla*_CTX-M-15_		*E. coli*	1		ND	ND	
Mixed green vegetables	*bla*_CTXM-15_	2017	*E. cloacae*	1	Canada	*bla*_TEM-1_, qnrB1, aac(6′) Ib cr	ND	[55]
Sambhar vegetables	*bla*_CTXM15_, *bla*_SHV-106_		*K. pneumoniae*	1		ND	ST101	
Aster scaber	*bla*_CTX-14_, *bla*_TEM-1_	2018	*E. coli*	1	South Korea	ND	ST69	[51]
Perilla leaf	*bla*_CTX-M-27_, *bla*_TEM-1_			1		ND	ST349	
Sweet potato stalk	*bla*_CTX-M-15_			1		ND	ST224	
Pepper leaf	*bla*_CTX-M-55_, *bla*_TEM-1_			1		ND	ND	
Mapleleaf ainsliaea	*bla*_CTX-M-27_			1		ND	ST10	
Leafy greens	*bla*_CTX-M_	2015–2016	*Enterobacterale*	1	United States	ND	ND	[53]
	* bla *_ CMY _		*Enterobacterale*	6		ND	ND	
Frisee salad	*bla*_CTX-M-1_, *bla*_DHA-1_	2015–2016	*E. cloacae*	2	Italy	ND	ND	[43]
Frisee salad + carrot	*bla*_CTX-M-15_		*Pantoea agglomerans*	6		ND	ND	
Peach	*bla*_CTX-M-15_	2013–2014	*K. pneumoniae*	1	Algeria	aadA2, strB, strA, qnrS1, oqxB, oqxA, fosA, mph(A), catA2, sul1, sul2, dfrA12	ST219	[48]

## 6. Vegetables and Fruit Isolates with Carbapenemase Genes

Only eight reports have revealed the isolation of Gram-negative bacteria producing carbapenemase genes from vegetables and fruit (Figure 1, Table 2). The first study describing carbapenemase-producing Gram-negative bacteria from fresh vegetable samples was published in 2015. Samples were purchased from different retail shops specializing in Asian food from three different cities in Switzerland, imported from Vietnam, Thailand, and India. In this study, only one *Klebsiella variicola* strain carrying the *bla*_OXA-181_ gene was isolated from a coriander sample from Thailand/Vietnam, and the obtained isolate coharbored a quinolone resistant gene (qnrS1). These data suggest that the international production of imported fresh vegetables constitutes a possible reservoir for the spread of carbapenemase-producing Gram-negative bacteria, especially Enterobacteriaceae [57].

Since its first report, carbapenemase genes have been identified in bacteria from different vegetable samples in only five countries across the Asian, African and European continents. Indeed, OXA-72-producing *Acinetobacter calcoaceticus* strains have been found in two vegetable samples purchased from the same market in Beirut, Lebanon [58]. In Japan, two *K. pneumoniae* and one *Acinetobacter baumannii* isolate were collected from vegetable samples in the city of Higashi-Hiroshima. Both *K. pneumoniae* isolates carried *bla*_NDM-1_ with other genes conferring resistance to β-lactams (*bla*_CTX-M-15_, *bla*_OXA-9_, and *bla*_TEM-1A_), aminoglycosides (*aac(6’)-Ib*, *aadA1*, and *aph(3’)-VI*), quinolones (*qnrS1*) and fluoroquinolones (*aac(6’)-Ib-cr*). While the obtained *A. baumannii* isolate coharbored *bla*_OXA-66_, *bla*_OXA-72_ and genes conferring resistance to sulfonamides (*sul2*), tetracycline (*tet*(B)), and streptomycin (*strAB*) [59]. In China, Wang et al., identified an *Escherichia coli* strain coproducing *bla*_KPC-2_ and *bla*_NDM-1_ genes in fresh lettuce from a market in Guangzhou. In addition, this multidrug resistant *E. coli* strain coharbored fosfomycin resistance genes (fosA3 and floR). This study represents the first report of either *bla*_KPC_ or *bla*_NDM_ genes in bacteria obtained from vegetables [60]. Additionally, two other studies from China reported the detection of carbapenemase-positive enterobacterial isolates. The first reported the isolation of twelve carbapenem-resistant isolates obtained from vegetable samples, where the highest detection rate was found in curly endive samples. The authors identified two *K. pneumoniae* isolates carrying the *bla*_KPC-2_ gene, while five of each *E. coli* and *C. freundii* strains harbored the *bla*_NDM_ gene, including four *E. coli* with the *bla*_NDM-5_ gene and five *C. freundii* with *bla*_NDM-1_. Notably, one *E. coli* strain from a cucumber sample harbored *bla*_NDM-5_ and *bla*_KPC-2_ genes simultaneously. All *C. freundii* and *E. coli* isolates carried fosfomycin resistance genes (fosA3and floR), and all *K. pneumoniae* and *C. freundii* isolates harbored the floR gene. However, one strain of *E. coli* and *C. freundii* harbored the aminoglycoside resistance gene (rmtB). Quinolone resistance genes including oqxAB and qnrB, were found in four and eight isolates, respectively [61]. The second report signaled the detection of two *E. coli* isolates carrying *bla*_NDM_ genes in leaf rape and spinach recovered from two supermarkets in Shandong province; one isolate concomitantly harbored *bla*_NDM-9_, mcr-1 and fosA3, while the second isolate carried *bla*_NDM-5_, mcr-1 and fosA3 genes [4].

From the African continent only one study from Algeria has been reported. The authors identified three *K. pneumoniae* isolates harboring the *bla*_OXA-48_ gene from lettuce, tomatoes and parsley in Béjaïa city [11]. In Europe and more precisely from Romania, the *bla*_OXA-48_ and *bla*_KPC_ genes were detected in *E. cloacae* and *K. oxytoca* isolates from parsley samples [46].

**Table 2:** Carbapenemases and mcr genes reported in Gram-negative bacteria isolates from vegetables and fruit worldwide.

Vegetables Type	Carbapenemase/mcr Gene	Isolation Period	Species	Isolates Number	Country	Other Antibiotic Resistance Genes	Sequence Type	Plasmid Type	Reference
Coriander	*bla*_OXA-181_	2015	*Klebsiella variicola*	1	Switzerland	qnrS1	ND	IncX3	[57]
Lettuce	*bla*_KPC-2_ and *bla*_NDM-1_	2015	*Escherichia coli*	1	China	*bla*_DHA-1_, fosA3, floR aacA4, tet(D), sul1, armA, mph(E), msr(E), erm(B), strA, strB	ST877	IncA/C (*bla*_NDM-1_), Untypable (*bla*_KPC-2_)	[60]
	*bla*_OXA-48_	2016	*K. pneumoniae*	1	Algeria	*bla*_TEM-1_	ST391	ND	[11]
	*bla*_NDM-5_	2017	*E. coli*	1	China	*bla*_CTX−M−1G_, fosA3, floR	ST4762	X3	[61]
				1		*bla*_CTX−M−1G_, fosA3, floR, oqxAB.	ST4762	X3	
	*bla*_NDM-1_		*C. freundii*	1		fosA3, floR, qnrB	ND	ND	
Parsley	*bla*_NDM-1_	2015	*K. pneumoniae*	1	Japan	*bla*_SHV-28_, *bla*_SHV-1_, *bla*_TEM-1A_, *bla*_CTX-M-15_, *bla*_CTX-M-14b_, *bla*_TEM-1A_, *bla*_OXA-9_, fosA, oqxAB, tet(D), aac(69)-Ib, aadA1, aph(39)-VI, aph(6)-Id, aph(39)-VIb, aph(39’)-Ib, aac(69)-Ib-cr, qnrS1	ST15	ND	[59]
	*bla*_OXA-48_	2016	*K. pneumoniae*	1	Algeria	None	ND	ND	[11]
	*bla*_OXA-48_	2019	*E. cloacae*	1	Romania	ND	ND	ND	[46]
	*bla*_KPC_		*K. oxytoca*	1		ND	ND	ND	
Baby leaf mix	*bla*_NDM-1_	2015	*K. pneumoniae*	1	Japan	*bla*_CTX-M-15_, *bla*_OXA-9_, *bla*_TEM-1A_, *bla*_SHV-28_, *_bla_*_CTX-M-14b_, fosA, oqxAB, aac(69)-Ib, aadA1, aph(39)-VI, aac(69)-Ib-cr, qnrS1, aph(6)-Id, aph(39)-VIb, aph(39’)-Ib.	ST15	ND	[59]
	*bla*_OXA-66_, *bla*_OXA-72_		*A. baumannii*	1		*bla*_ADC-25_, *bla*_OXA-66_, *bla*_OXA-72_, sul2, tet(B), aac(3)-Ia aac(69)-Ip, aph(39’)-Ib, aph(6)-Id	ST2	GR2 (*bla*_OXA-72_)	
Cucumber	*bla*_KPC-2_	2017	*K. pneumoniae*	1	China	qnrB, oqxAB	ST23	F35:A-:B1	[61]
				1		*bla*_CTX−M−1G_, qnrB, oqxAB	ST23	F35:A-:B1	
	*bla*_NDM-5_		*E. coli*	1		fosA3, floR, qnrB	UT	ND	
				1		*bla*_CTX−M−1G_, fosA3, floR,.	ST4762	ND	
	*bla*_NDM-5_ and *bla*_KPC-2_		*E. coli*	1		None	ND	ND	
Curly endive	*bla*_NDM-5_	2017	*E. coli*	1	China	*bla*_CTX−M−1G_, fosA3, floR, rmtB	ST167	ND	[61]
	*bla*_NDM-1_		*C. freundii*	1		fosA3, floR, qnrB	ND	ND	
				1		fosA3, floR, oqxAB, qnrB, rmtB	ND	ND	
				1		fosA3, floR, qnrB	ND	X3	
Tomato	*bla*_OXA-48_	2016	*K. pneumoniae*	1	Algeria	None	ST1877	ND	[11]
	*bla*_NDM-1_	2017	*C. freundii*	1	China	*bla*_CTX−M−1G_, fosA3, floR, qnrB	ND	X3	[61]
Leaf rape	*bla*_NDM-5_	2017–2018	*E. coli*	1	China	mcr-1, fosA3	ST156	X3	[4]
Spinach	*bla*_NDM-9_			1		mcr-1, fosA3	ST2847	Untypable	
	*bla*_KPC_	2019	*Morganella morganii*	1	Romania	ND	ND	ND	[46]
Vegetables (ND)	*bla*_OXA-72_	ND	*Acinetobacter calcoaceticus*	2	Lebanon	ND	ND	ND	[58]
Lettuce	mcr-1	2013	*E. coli*	1	Portugal	*bla*_TEM-1_, *aadA1y*, *aph(4)-Ia*, *estX-12*, *floR*, *sat2*, *strA*, *strB*, *sul2*, *tetA*	ST1716	ND	[62]
		2013–2014	*E. coli*	1		*bla*_TEM-1_, aac(3)-Iv, aadA1, aph(4)-Ia, aph(6)-Id, mdf(A)-type, tetA, sul2, floR	ST1716	ND	[63]
		2016	*E. coli*	1	China	*bla*_CTX-M-14_, floR, fosA3, oqxAB	ST795	IncHI2	[64]
				1		*bla*_CTX-M-55_, floR	ST2505	ncI2	
		2015	*E. coli*	1		*bla*_CTX-M-55_, rmtB, floR, fosA3.	ST156	IncI2	
		2016	*E. coli*	1		floR	ST48	IncX4	
		2015	*Raoultella ornithinolytica*	2		*bla*_CTX-M-14_, floR, fosA3, oqxAB	NA	IncHI2	
	mcr-1	2018	*E. coli*	1	South Korea	*bla*_TEM-1_ and *bla*_CTX-M-55_	ST10	ND	[65]
	mcr-1	2017–2018	*E. coli*	1	China	ND	ST10	X4	[66]
				1		ND	ST2705	HI2	
Tomato	mcr-1	2016	*E. coli*	1	China	*bla*_CTX-M-14_, floR, fosA3, oqxAB	ST69	IncHI2	[64]
		2015	*E. coli*	2		floR	ST206	chromosome	
	mcr-1	2017–2018	*E. coli*	1	China	ND	ST713	X4	[66]
				1	China	ND	UT	I2	
Leaf rape	mcr-1	2017–2018	*K. pneumoniae*	1	China	*bla*_NDM-5_, fosA3	ST156	X4	[4]
			*E. coli*	1	China	ND	ST744	X4	[66]
Green Pepper	mcr-1	2017–2018	*E. cloacae*	1	China	ND	ND	ND	[66]
	mcr-1		*E. coli*	1		ND	ST5873	X4	
Spinach	mcr-1	2017–2018	*E. coli*	1	China	*bla*_NDM-9_, fosA3	ST2847	I2	[4]
	mcr-1			1		ND	ST2253	I2	[66]
Cha-om	mcr-1	2014	*E. coli*	1	Switzerland	*bla*_CTX-M-55_	ST167	ND	[67]
Basil leaves	mcr-1	2014	*E. coli*	1		*bla*_CTX-M-65_	ST4683	ND	
Cucumber	mcr-1	2017–2018	*E. coli*	2	China	ND	ST744	X4	[66]
				2		ND	ST1115	I2	
Carrot	mcr-1	2017–2018	*E. coli*	1		ND	ST5539	X4	
			*E. coli*	1		ND	ST13	I2	
Curly endive	mcr-1	2017–2018	*E. coli*	1		ND	ST13	X4	
Pak choi	mcr-1	2017–2018	*E. coli*	1		ND	ST648	I2	
Apple	mcr-1	2016	*E. coli*	1	China	aadA2, aadA1,floR, cmlA1, sul2, sul3, tetA, tetM, dfrA12,mdfA	ST189	IncFIA	[68]
Orange	mcr-1		*K. pneumoniae*	1		*bla*_SHV-110_, qnrS1, oqxA, oqxB, fosA6, sul1, tetA, dfrA1	ST442	IncHI1	

## 7. Vegetables and Fruit Isolates with the mcr Gene

To date, eight studies have reported mcr-producing Gram-negative bacteria, especially isolates of Enterobacteriaceae species, from fresh produce that mostly originated from China (Figure 1, Table 2). The mcr-1 gene was first reported in 2016 in Switzerland in two out of sixty isolates. The two *E. coli* isolates carried the mcr-1 gene and coharbored *bla*_CTX-M-55_ and *bla*_CTX-M-65_ genes, respectively [67]. After this first description in fresh produce, mcr-1-producing GNB isolates on fresh produce were reported in China, where seven *E. coli* and two *Raoultella ornithinolytica* isolates were recovered from tomato and lettuce samples between May 2015 and August 2016 in Guangzhou. All the obtained mcr-1-positive strains harbored the florfenicol resistance gene (floR). Of the nine isolates, six strains carried the *bla*_CTX-M_ gene (four *bla*_CTX-M-14_ and two *bla*_CTX-M-55_), with five and four strains harboring the fosA3 and oqxAB efflux pump gene, respectively [64]. Moreover, the mcr-1 gene was described in China from a total of 528 fresh vegetable samples, including 18 different types purchased from 53 supermarkets and farmers markets from 23 districts or cities in nine provinces between May 2017 and April 2018. Of the 528 samples analyzed, only 19 samples harbored one or more mcr-positive isolates, and the three highest detection rates were noted in carrots (14.3%), pakchoi (13.3%) and green pepper (7.7%), followed by leaf lettuce (5.6%), leaf rape (4.9%), romaine lettuce (4.3%), tomato (3.5%), spinach (3.2%), cucumber (3.1%), and curly endive (2.4%). In the above study, twenty-four mcr-1-positive isolates were obtained; twenty-three strains were identified as *E. coli* and one as *E. cloacae*. Fourteen mcr-1-positive strains coproduced the *bla*_CTX-M_ gene, nine strains harbored the *bla*_CTX-M-9G_ gene and three strains carried *bla*_CTX-M-1G_. However, the remaining two strains harbored both *bla*_CTX-M-9G_ and *bla*_CTX-M-1G_ genes. In addition, eight and two isolates harbored fosA3 and rmtB genes, respectively. Plasmid-mediated resistance to quinolones (PMQR), including oqxAB, qnrS and qnrB genes, were also detected in this study [66]. Additionally, in the same country, two *E. coli* isolates carrying the mcr-1 gene were isolated from leaf rape and spinach in Shandong province. These isolates coharbored metallo-β-lactamase and fosA3 genes; the first carried *bla*_NDM-5_, while the second harbored the *bla*_NDM-9_ gene [4]. In 2018, one *E. coli* isolate carrying the mcr-1 gene recovered from lettuce was reported in South Korea. The obtained isolate coharbored *bla*_TEM-1_ and *bla*_CTX-M-55_ genes [65]. In Portugal, the mcr-1 gene was reported by Manageiro et al. in 2020, and they documented the presence of this gene in an *E. coli* strain isolated from a lettuce sample. In silico analysis showed the presence of additional antibiotic resistance genes including *bla*_TEM-1_, aac(3)-Iv, aadA1, aph(4)-Ia, aph(6)-Id, mdf(A)-type, tetA, sul2 and floR-type [63]. Another report from Portugal revealed the detection of the mcr-1 gene in an *E. coli* strain from conventionally produced lettuce. The isolate co-carried *bla*_TEM-1_, aph(4)-Ia, floR, sat2, strA, strB, sul2 and tetA genes, while no conventional and organic fruit were positive for the mcr-1 gene [62].

On fruit samples, the mcr-1 gene was detected in *E. coli* and *K. pneumoniae* isolates from apple and orange samples recovered in China [68].

## 8. Contamination Pathways and Genetic Characteristics of β-Lactamases and mcr-Producing Gram-Negative Bacteria

The high diversity of global clones illustrates the extensive spread of ESBL-producing *K. pneumoniae* and *E. coli* isolates on vegetables around the world (ST45, ST219, ST15 and ST147 found in *K. pneumoniae* isolates, and ST410-A, ST44, ST405, ST131 and ST38 in *E. coli* isolates). In Algeria, sequence type 14, ST45, ST219, ST236, and ST882 have been identified among *K. pneumoniae* strains recovered on fresh fruit and vegetables carrying ESBL or cephalosporinase genes, including *bla*_CTX-M-15_, *bla*_OXA-1_, *bla*_SHV-101_, *bla*_SHV-28_ and *bla*_DHA-1_ genes that were mostly (11 of 13) located on the IncFII plasmid, while the IncR plasmid replicon was identified in only one isolate [48]. In Switzerland, twenty-two different sequence types identified in *E. coli*-positive ESBL have been described on imported vegetables, including ST4684 and ST4683, four of them belonging to the epidemiologically important sequence types ST405 (*n* = 1), ST131 (*n* = 2) and ST38 (*n* = 2) [10]. Similarly, the ST131 *E. coli* clone is known for its role in the global spread of ESBLs, especially CTX-M-15, and this clone has had an inevitable clinical impact on antibiotic resistance and pathogenicity [69]. In the same study conducted in Switzerland, high clonal diversity was observed among *K. pneumoniae* strains, with two isolates belonging to the epidemic clones ST15 and ST147 [10]. In Quito, Ecuador, the hyper epidemic clones ST410 and ST44 harboring the *bla*_CTX-M-15_ gene have been identified in *E. coli* isolates from leaf lettuce, alfalfa and parsley/cilantro, and three of them were found on the same integron 1 variable region (dfrA1 and addA5). The five remaining isolates presented *bla*_CTX-M-15_ downstream of an insertion sequence element p1 (ISEcp1) [54]. In a survey in Germany, a high diversity of global clones was identified in ESBL-producing *E. coli* isolates from different vegetable samples. These ESBL determinants were detected on different plasmids as follows: IncHI2 and IncK (*bla*_CTX-M-14_, ST527, ST973), IncN (*bla*_CTX-M-65_, ST10), IncFIB (*bla*_CTX-M-15_, ST410), IncHI2 (*bla*_CTX-M-125_, ST542) and IncFIA-FIB (*bla*_CTX-M-2_, ST120) [45]. The IncFIB and IncFIC plasmid replicons were found in *Pseudomonas hunanensis,* and *P. putida* carried the *bla*_SHV-12_ respectively.The plasmid IncK/B was reported in *P. paralactis* harboring the *bla*_TEM-16_ gene [49].

The dissemination of carbapenemase-producing *E. coli* is polyclonal, where multiple STs have been reported. The sequence type 877 was reported in an *E. coli* strain coproducing *bla*_KPC-2_ and *bla*_NDM-1_ genes isolated from fresh lettuce. These genes were located on 64 and 118 kb plasmids, designated as plasmids pHNTS79-KPC and pHNTS79-NDM, respectively [60]. Furthermore, ST1877 and ST391 have been detected in OXA-48-positive *K. pneumoniae* isolates from lettuce, tomatoes and parsley in Algeria, where the ST391 clone is considered as an emergent carbapenemase-producing lineage of clinical importance [11]. The *bla*_OXA-181_ detected in a *Klebsiella variicola* isolate from a coriander sample was mediated by the IncX3-type plasmid of 51-kb [57]. Indeed, in Japan the epidemic clones ST15 and ST2 were reported among *K. pneumoniae* that carried the *bla*_NDM-1_ gene and the *A. baumannii* strain coharbored *bla*_OXA-66_, and *bla*_OXA-72_ genes, respectively [59]. ST15 is a relatively common NDM-positive *K. pneumoniae* lineage, and it has been found in various countries across different continents, almost all of which were isolated from humans [70]. IncX3 plasmids carrying the *bla*_NDM_ gene have been identified in *E. coli* and *C. freundii* strains isolated from cucumbers, and the identified IncX3 plasmid was identical or highly similar (99%) to the IncX3 plasmids identified from patients in other countries. In addition, similar F35:A-:B1 plasmids were described in two *bla*_KPC-2_-producing-*K. pneumoniae* isolates belonging to ST23 obtained from different cities. Two *E. coli* isolates carrying the *bla*_NDM-5_ gene isolated from cucumber and romaine lettuce samples in different cities in China shared an identical PFGE pattern and sequence type (ST4762); however, one *E. coli* strain belonged to ST167 [61].

Among *E. coli* isolates, the mcr-1 gene was found in multiple STs from different countries, ST10 in South Korea, and ST167 and ST4683 in Switzerland [65,67]. ST156 and ST2847 have been identified in China from leaf rape and spinach samples. In the latter, mcr-1 genes were located on the~60-kb IncI2 plasmid or the ~33-kb IncX4 plasmid; even as *bla*_NDM-5_ was on the ~46-kb IncX3 plasmid while *bla*_NDM-9_ was on the ~120-kb untypeable plasmid. The detected plasmids were highly similar to those from patients and animals described in different countries [4]. In a Chinese study, six sequence types, including ST795, ST2505, ST69, ST156, ST48 and ST206, were described in seven *E. coli* isolates recovered from lettuce and tomato samples. For the four *E. coli* isolates, mcr-1 genes were located on IncHI2, IncI2 or IncX4 plasmids, while for the two *Raoultella ornithinolytica* isolates, the mcr-1 gene was located on the IncHI2 plasmid [64]. Moreover, in China, sixteen STs along with a new ST type were identified, while the most prevalent STs were ST744 and ST224. In this study, different plasmid replicons were detected, including IncX4, IncI2 and IncHI2; where IncX4 was the most detected and shared highly similar RFLP profiles, although they were from different cities and fresh vegetables. The mcr-1 gene was located on the IncX4 plasmid of ∼33 kb in size [66].

In addition to the above results, the study found that patients and animals shared identical or highly similar plasmids with vegetables [4,61]. Various other studies reported that vegetables may become contaminated with multidrug resistant bacteria from soil, manure fertilization, irrigation water or through direct contamination by humans [2]. In this context, the major way in which antibiotic resistance enters the soil is through the use of animal manure [71]. In Australia, Zhang et al. explored the impact of cattle and poultry manure application on the resistome in lettuce and soil microbiomes, including the rhizosphere, root endosphere, leaf endosphere and phyllosphere. In addition, they identified potential transmission routes of antibiotic resistance genes in the soil–plant system. The authors reported that poultry manure application increased antibiotic resistance genes in the rhizosphere, root endophyte and phyllosphere, while cattle manure use increased the abundance of antibiotic resistance genes only in the root endophyte, suggesting that poultry manure may have a stronger impact on lettuceresistomes. Moreover, the authors also identified an overlap of antibiotic resistance gene (ARG) profiles between lettuce tissues and soil, which indicates that plant and environmental resistomes are interconnected, and confirmed the transmission of antibiotic resistance genes from manured soil to vegetables. Two main transmission pathways were reported: an internal pathway through plant tissues and an external pathway via aerosol from the atmosphere to the plant surface. Thus, in the external pathway, sixty-nine ARGs were shared between poultry manure-amended soil and the phyllosphere of lettuce, while in the internal pathway 47 genes were common between rhizosphere soil and the root endophyte [72]. This finding was consistent with previous studies which reported that the phyllosphere resistome was significantly more abundant and diverse than the endophytic resistome in organic vegetables [73,74]. Another report from China described the impact of the long-term use of inorganic (chemical) and organic (manure) fertilizers on antibiotic resistance genes in greenhouse soils growing vegetables. The results showed that both inorganic and organic use increased the abundance and the diversity of soil ARGs, with a difference in the dominant ARG types. ARG abundance and diversity were both higher in organic fertilizer [75]. These data confirmed those of previous reports indicating that fertilizer application, especially organic fertilizer such as animal manure, raised ARG abundance and diversity in soil compared to soil without fertilization [75,76,77].

Several studies have reported food-borne human outbreaks linked to the consumption of fresh vegetables and fruit irrigated with wastewater and indicated that the type of irrigation practice plays a vital role in the contamination of farm produce [78]. In this context, Araújo et al. characterized the presence of *E. coli* isolates on vegetables and in irrigation water sampled from 16 household farms in Portugal. In this later study, different commonly acquired genes such as *bla*_TEM_, *tetA* and *tetB* and plasmids (IncFIC, IncFrep and IncFIB) were detected in isolates in both water and vegetable samples. In addition, rep-PCR typing results detected the same STs and identical clones in vegetables and water, suggesting cross-contamination. These results suggest that irrigation groundwater is a reservoir of antibiotic resistant *E. coli* and may enter the food chain via vegetable consumption [79]. Makkaew et al. evaluated the contamination of lettuce by *E. coli*, grown under four diverse methods of wastewater irrigation: open spray, open drip, spray under plastic sheet cover, and drip under plastic sheet cover. *E. coli* contamination was reported in all lettuce samples in both open and covered spray beds in all types of spray beds. An equal level of microbial quality of spray bed lettuce and submersed lettuce irrigated with wastewater containing 1299.7 *E. coli* MPN/100 mL was detected, and this result was similar in both laboratory and experimental investigations [80]. In Ghana, Antwi-Agyei et al. reported that irrigation with partially treated and untreated wastewater is a key risk factor for the observed contamination of 80% of produce samples, with a median concentration ranging from 0.64 to 3.84 log *E. coli*/g produce, while ready-to-eat salad was the most contaminated with 4.23 log *E. coli*/g [81].

## 9. Conclusions

This review provides a reference for an enhanced understanding of the global risk of fresh vegetables and fruit in the transmission of multidrug resistant Gram-negative bacteria and emphasizes the necessity of paying close attention to these products as a future public health issue. Given that fresh produce is often consumed raw, this allows the transfer of these antibiotic resistance genes to human gut bacteria. It is now even more important that more investigations should be performed in order to survey the emergence and transmission of these genes to humans from farm to fork. In addition, suitable measures, including the improvement of water quality and agricultural practices, need to be considered to ensure consumer safety worldwide.

## References

[1] Xylia P., Botsaris G., Chrysargyris A., Skandamis P., Tzortzakis N. (2019). Variation of microbial load and biochemical activity of ready-to-eat salads in Cyprus as affected by vegetable type, season, and producer. Food Microbiol..

[2] Holzel C.S., Tetens J.L., Schwaiger K. (2018). Unraveling the role of vegetables in spreading antimicrobial-resistant bacteria: A need for quantitative risk assessment. Foodborne Pathog. Dis..

[3] Van Hoek A.H., Veenman C., Van Overbeek W.M., Lynch G., De Roda Husman A.M., Blaak H. (2015). Prevalence a characterization of ESBL- and AmpC-producing Enterobacteriaceae on retail vegetables. Int. J. Food Microbiol..

[4] Liu B.T., Song F.J. (2019). Emergence of two *Escherichia coli* strains co-harboring mcr-1 and *bla*
_NDM_ in fresh vegetables from China. Infect. Drug Resist..

[5] Doi Y., Paterson D.L. (2015). Carbapenemase-producing Enterobacteriaceae. Semin. Respir. Crit. Care Med..

[6] Bassetti M., Pecori D., Sibani M., Corcione S., De Rosa F.G. (2015). Epidemiology and treatment of MDR Enterobacteriaceae. Curr. Treat. Options Infect. Dis..

[7] Ye Q., Wu Q., Zhang S., Zhang J., Yang G., Wang J., Xue L., Chen M. (2018). Characterization of extended spectrum beta-lactamase-producing Enterobacteriaceae from retail food in China. Front. Microbiol..

[8] Bakthavatchalam Y.D., Pragasam A.K., Biswas I., Veeraraghavan B. (2018). Polymyxin susceptibility testing, interpretative breakpoints and resistance mechanisms: An update. J. Glob. Antimicrob. Resist..

[9] Richter L., Du Plessis E.M., Duvenage S., Korsten L. (2019). Occurrence, identification, and antimicrobial resistance profiles of Extended-Spectrum and AmpC beta-Lactamase-producing Enterobacteriaceae from fresh vegetables retailed in Gauteng Province, South Africa. Foodborne Pathog. Dis..

[10] Zurfluh K., Nuesch-Inderbinen M., Morach M., Zihler B.A., Hachler H., Stephan R. (2015). Extended-spectrum-beta-lactamase-producing Enterobacteriaceae isolated from vegetables imported from the Dominican Republic, India, Thailand, and Vietnam. Appl. Environ. Microbiol..

[11] Touati A., Mairi A., Baloul Y., Lalaoui R., Bakour S., Thighilt L., Gharout A., Rolain J.M. (2017). First detection of *Klebsiella pneumoniae* producing OXA-48 in fresh vegetables from Bejaia city, Algeria. J. Glob. Antimicrob. Resist..

[12] Bush K., Bradford P.A. (2020). Epidemiology of beta-Lactamase-producing pathogens. Clin. Microbiol. Rev..

[13] Nordmann P. (2014). Carbapenemase-producing Enterobacteriaceae: Overview of a major public health challenge. Med. Mal. Infect..

[14] Bush K. (2018). Past and present perspectives on beta-Lactamases. Antimicrob. Agents Chemother..

[15] Nordmann P., Poirel L. (2019). Epidemiology and diagnostics of carbapenem resistance in Gram-negative bacteria. Clin. Infect. Dis..

[16] Nordmann P., Dortet L., Poirel L. (2012). Carbapenem resistance in Enterobacteriaceae: Here is the storm!. Trends Mol. Med..

[17] Sawa T., Kooguchi K., Moriyama K. (2020). Molecular diversity of extended-spectrum beta-lactamases and carbapenemases, and antimicrobial resistance. J. Intensive Care.

[18] Meini S., Tascini C., Cei M., Sozio E., Rossolini G.M. (2019). AmpC beta-lactamase-producing Enterobacterales: What a clinician should know. Infection.

[19] Hennequin C., Ravet V., Robin F. (2018). Plasmids carrying DHA-1 beta-lactamases. Eur. J. Clin. Microbiol. Infect. Dis..

[20] Doi Y. (2019). Treatment options for carbapenem-resistant Gram-negative bacterial infections. Clin. Infect. Dis..

[21] Yigit H., Queenan A.M., Anderson G.J., Domenech-Sanchez A., Biddle J.W., Steward C.D., Alberti S., Bush K., Tenover F.C. (2001). Novel carbapenem-hydrolyzing beta-lactamase, KPC-1, from a carbapenem-resistant strain of *Klebsiella pneumoniae*. Antimicrob. Agents Chemother..

[22] Elshamy A.A., Aboshanab K.M. (2020). A review on bacterial resistance to carbapenems: Epidemiology, detection and treatment options. Future Sci. OA.

[23] Yong D., Toleman M.A., Giske C.G., Cho H.S., Sundman K., Lee K., Walsh T.R. (2009). Characterization of a new metallo-beta-lactamase gene, bla(NDM-1), and a novel erythromycin esterase gene carried on a unique genetic structure in *Klebsiella pneumoniae* sequence type 14 from India. Antimicrob. Agents Chemother..

[24] Lupo A., Papp-Wallace K.M., Sendi P., Bonomo R.A., Endimiani A. (2013). Non-phenotypic tests to detect and characterize antibiotic resistance mechanisms in Enterobacteriaceae. Diagn. Microbiol. Infect. Dis..

[25] Stefaniuk E.M., Tyski S. (2019). Colistin resistance in Enterobacterales strains—A current view. Pol. J. Microbiol..

[26] Lima T., Domingues S., Da Silva G.J. (2019). Plasmid-mediated colistin resistance in *Salmonella enterica*: A review. Microorganisms.

[27] Gharaibeh M.H., Shatnawi S.Q. (2019). An overview of colistin resistance, mobilized colistin resistance genes dissemination, global responses, and the alternatives to colistin: A review. Vet. World.

[28] Mendes Oliveira V.R., Paiva M.C., Lima W.G. (2019). Plasmid-mediated colistin resistance in Latin America and Caribbean: A systematic review. Travel. Med. Infect. Dis..

[29] Kai J., Wang S. (2019). Recent progress on elucidating the molecular mechanism of plasmid-mediated colistin resistance and drug design. Int. Microbiol..

[30] Anyanwu M.U., Jaja I.F., Nwobi O.C. (2020). Occurrence and characteristics of mobile colistin resistance (mcr) gene-containing isolates from the environment: A review. Int. J. Environ. Res. Public Health.

[31] Liu Y.Y., Wang Y., Walsh T.R., Yi L.X., Zhang R., Spencer J., Doi Y., Tian G., Dong B., Huang X. (2016). Emergence of plasmid-mediated colistin resistance mechanism MCR-1 in animals and human beings in China: A microbiological and molecular biological study. Lancet Infect. Dis..

[32] Hinchliffe P., Yang Q.E., Portal E., Young T., Li H., Tooke C.L., Carvalho M.J., Paterson N.G., Brem J., Niumsup P.R. (2017). Insights into the mechanistic basis of plasmid-mediated colistin resistance from crystal structures of the catalytic domain of MCR-1. Sci. Rep..

[33] Xavier B.B., Lammens C., Ruhal R., Kumar-Singh S., Butaye P., Goossens H., Malhotra-Kumar S. (2016). Identification of a novel plasmid-mediated colistin-resistance gene, mcr-2, in *Escherichia coli*, Belgium, June 2016. Eurosurveillance.

[34] Yin W., Li H., Shen Y., Liu Z., Wang S., Shen Z., Zhang R., Walsh T.R., Shen J., Wang Y. (2017). Novel plasmid-mediated colistin resistance gene mcr-3 in *Escherichia coli*. MBio.

[35] Carattoli A., Villa L., Feudi C., Curcio L., Orsini S., Luppi A., Pezzotti G., Magistrali C.F. (2017). Novel plasmid-mediated colistin resistance mcr-4 gene in *Salmonella* and *Escherichia coli*, Italy 2013, Spain and Belgium, 2015 to 2016. Eurosurveillance.

[36] Borowiak M., Fischer J., Hammerl J.A., Hendriksen R.S., Szabo I., Malorny B. (2017). Identification of a novel transposon-associated phosphoethanolamine transferase gene, mcr-5, conferring colistin resistance in d-tartrate fermenting *Salmonella enterica* subsp. enterica serovar Paratyphi B. J. Antimicrob. Chemother..

[37] AbuOun M., Stubberfield E.J., Duggett N.A., Kirchner M., Dormer L., Nunez-Garcia J., Randall L.P., Lemma F., Crook D.W., Teale C. (2018). mcr-1 and mcr-2 (mcr-6.1) variant genes identified in Moraxella species isolated from pigs in Great Britain from 2014 to 2015. J. Antimicrob. Chemother..

[38] Yang Y.Q., Li Y.X., Lei C.W., Zhang A.Y., Wang H.N. (2018). Novel plasmid-mediated colistin resistance gene mcr-7.1 in *Klebsiella pneumoniae*. J. Antimicrob. Chemother..

[39] Wang X., Wang Y., Zhou Y., Li J., Yin W., Wang S., Zhang S., Shen J., Shen Z., Wang Y. (2018). Emergence of a novel mobile colistin resistance gene, mcr-8, in NDM-producing *Klebsiella pneumoniae*. Emerg. Microbes Infect..

[40] Carroll L.M., Gaballa A., Guldimann C., Sullivan G., Henderson L.O., Wiedmann M. (2019). Identification of novel mobilized colistin resistance gene mcr-9 in a multidrug-resistant, colistin-susceptible *Salmonella enterica Serotype Typhimurium* isolate. MBio.

[41] Wang C., Feng Y., Liu L., Wei L., Kang M., Zong Z. (2020). Identification of novel mobile colistin resistance gene mcr-10. Emerg. Microbes Infect..

[42] Blaak H., Van Hoek A.H., Veenman C., Docters Van Leeuwen A.E., Lynch G., Van Overbeek W.M., De Roda Husman A.M. (2014). Extended spectrum β-lactamase- and constitutively AmpC-producing Enterobacteriaceae on fresh produce and in the agricultural environment. Int. J. Food Microbiol..

[43] Iseppi R., De N.S., Bondi M., Messi P., Sabia C. (2018). Extended-spectrum beta-lactamase, AmpC, and MBL-producing Gram-negative bacteria on fresh vegetables and ready-to-eat salads sold in local markets. Microb. Drug Resist..

[44] Nuesch-Inderbinen M., Zurfluh K., Peterhans S., Hachler H., Stephan R. (2015). Assessment of the prevalence of Extended-Spectrum beta-Lactamase-producing Enterobacteriaceae in ready-to-eat Salads, fresh-cut fruit, and sprouts from the Swiss market. J. Food Prot..

[45] Freitag C., Michael G.B., Li J., Kadlec K., Wang Y., Hassel M., Schwarz S. (2018). Occurrence and characterisation of ESBL-encoding plasmids among *Escherichia coli* isolates from fresh vegetables. Vet. Microbiol..

[46] Colosi I.A., Baciu A.M., Opris R.V., Peca L., Gudat T., Simon L.M., Colosi H.A., Costache C. (2020). Prevalence of ESBL, AmpC and carbapenemase-producing Enterobacterales isolated from raw vegetables retailed in Romania. Foods.

[47] Richter L., Du Plessis E.M., Duvenage S., Korsten L. (2020). Occurrence, phenotypic and molecular characterization of extended-spectrum- and AmpC- β-Lactamase producing Enterobacteriaceae isolated from selected commercial spinach supply chains in South Africa. Front. Microbiol..

[48] Mesbah Z.F., Granier S.A., Touati A., Millemann Y. (2020). Occurrence of third-generation cephalosporins-resistant *Klebsiella pneumoniae* in fresh fruits and vegetables purchased at markets in Algeria. Microb. Drug Resist..

[49] Usui M., Ozeki K., Komatsu T., Fukuda A., Tamura Y. (2019). Prevalence of extended-spectrum beta-lactamase-producing bacteria on fresh vegetables in Japan. J. Food Prot..

[50] Kurittu P., Khakipoor B., Aarnio M., Nykasenoja S., Brouwer M., Myllyniemi A.L., Vatunen E., Heikinheimo A. (2021). Plasmid-borne and chromosomal ESBL/AmpC genes in *Escherichia coli* and *Klebsiella pneumoniae* in global food products. Front. Microbiol..

[51] Song J., Oh S.S., Kim J., Shin J. (2020). Extended-spectrum β-lactamase-producing *Escherichia coli* isolated from raw vegetables in South Korea. Sci. Rep..

[52] Bhutani N., Muraleedharan C., Talreja D., Rana S.W., Walia S., Kumar A., Walia S.K. (2015). Occurrence of multidrug resistant extended spectrum beta-lactamase-producing bacteria on iceberg lettuce retailed for human consumption. BioMed Res. Int..

[53] Parker E., Albers A., Mollenkopf D., Korec D., Mathys D., Stuever D., Wittum T. (2021). AmpC- and Extended-Spectrum β-Lactamase-producing Enterobacteriaceae detected in fresh produce in central Ohio. J. Food Prot..

[54] Ortega-Paredes D., Barba P., Mena-Lopez S., Espinel N., Zurita J. (2018). *Escherichia coli* hyperepidemic clone ST410-A harboring *bla*_CTX-M-15_ isolated from fresh vegetables in a municipal market in Quito-Ecuador. Int. J. Food Microbiol..

[55] Jung D., Rubin J.E. (2020). Identification of antimicrobial resistant bacteria from plant-based food products imported into Canada. Int. J. Food Microbiol..

[56] Lopes R., Fuentes-Castillo D., Fontana H., Rodrigues L., Dantas K., Cerdeira L., Henriques I., Lincopan N. (2021). Endophytic lifestyle of global clones of Extended-Spectrum β-Lactamase-producing priority pathogens in fresh vegetables: A trojan horse strategy favoring human colonization?. MSystems.

[57] Zurfluh K., Poirel L., Nordmann P., Klumpp J., Stephan R. (2015). First detection of *Klebsiella variicola* producing OXA-181 carbapenemase in fresh vegetable imported from Asia to Switzerland. Antimicrob. Resist. Infect. Control.

[58] Al Atrouni A., Kempf M., Eveillard M., Rafei R., Hamze M., Joly-Guillou M.L. (2016). First report of Oxa-72-producing *Acinetobacter calcoaceticus* in Lebanon. New Microbes New Infect..

[59] Soliman A.M., Nariya H., Tanaka D., Yu L., Hisatsune J., Kayama S., Kondo K., Sugai M., Shimamoto T., Shimamoto T. (2021). Vegetable-derived carbapenemase-producing high-risk *Klebsiella pneumoniae* ST15 and *Acinetobacter baumannii* ST2 clones in Japan: Coexistence of *bla* (NDM-1), *bla* (OXA-66), *bla* (OXA-72), and an AbaR4-Like Resistance Island in the Same Sample. Appl. Environ. Microbiol..

[60] Wang J., Yao X., Luo J., Lv L., Zeng Z., Liu J.H. (2018). Emergence of *Escherichia coli* co-producing NDM-1 and KPC-2 carbapenemases from a retail vegetable, China. J. Antimicrob. Chemother..

[61] Liu B.T., Zhang X.Y., Wan S.W., Hao J.J., Jiang R.D., Song F.J. (2018). Characteristics of carbapenem-resistant Enterobacteriaceae in ready-to-eat vegetables in China. Front. Microbiol..

[62] Jones-Dias D., Manageiro V., Ferreira E., Barreiro P., Vieira L., Moura I.B., Canica M. (2016). Architecture of class 1, 2, and 3 integrons from Gram negative bacteria recovered among fruits and vegetables. Front. Microbiol..

[63] Manageiro V., Jones-Dias D., Ferreira E., Canica M. (2020). Plasmid-mediated colistin resistance (mcr-1) in *Escherichia coli* from non-imported fresh vegetables for human consumption in Portugal. Microorganisms.

[64] Luo J., Yao X., Lv L., Doi Y., Huang X., Huang S., Liu J.H. (2017). Emergence of mcr-1 in *Raoultella ornithinolytica* and *Escherichia coli* Isolates from retail vegetables in China. Antimicrob. Agents Chemother..

[65] Oh S.S., Song J., Kim J., Shin J. (2020). Increasing prevalence of multidrug-resistant mcr-1-positive *Escherichia coli* isolates from fresh vegetables and healthy food animals in South Korea. Int. J. Infect. Dis..

[66] Liu B.T., Li X., Zhang Q., Shan H., Zou M., Song F.J. (2019). Colistin-resistant mcr-positive Enterobacteriaceae in fresh vegetables, an increasing infectious threat in China. Int. J. Antimicrob. Agents.

[67] Zurfuh K., Poirel L., Nordmann P., Nuesch-Inderbinen M., Hachler H., Stephan R. (2016). Occurrence of the plasmid-borne mcr-1 colistin resistance gene in Extended-Spectrum-beta-Lactamase-producing Enterobacteriaceae in river water and imported vegetable samples in Switzerland. Antimicrob. Agents Chemother..

[68] Yang F., Shen C., Zheng X., Liu Y., El-Sayed Ahmed M.A.E., Zhao Z., Liao K., Shi Y., Guo X., Zhong R. (2019). Plasmid-mediated colistin resistance gene mcr-1 in *Escherichia coli* and *Klebsiella pneumoniae* isolated from market retail fruits in Guangzhou, China. Infect. Drug Resist..

[69] Chong Y., Shimoda S., Shimono N. (2018). Current epidemiology, genetic evolution and clinical impact of extended-spectrum β-lactamase-producing *Escherichia coli* and *Klebsiella pneumoniae*. Infect. Genet. Evol..

[70] Wu W., Feng Y., Tang G., Qiao F., McNally A., Zong Z. (2019). NDM Metallo-β-lactamases and their bacterial producers in health care settings. Clin. Microbiol. Rev..

[71] Pu C., Yu Y., Diao J., Gong X., Li J., Sun Y. (2019). Exploring the persistence and spreading of antibiotic resistance from manure to biocompost, soils and vegetables. Sci. Total Environ..

[72] Zhang Y.J., Hu H.W., Chen Q.L., Singh B.K., Yan H., Chen D., He J.Z. (2019). Transfer of antibiotic resistance from manure-amended soils to vegetable microbiomes. Environ. Int..

[73] Zhang Y.J., Hu H.W., Gou M., Wang J.T., Chen D., He J.Z. (2017). Temporal succession of soil antibiotic resistance genes following application of swine, cattle and poultry manures spiked with or without antibiotics. Environ. Pollut..

[74] Zhu B., Chen Q., Chen S., Zhu Y.G. (2017). Does organically produced lettuce harbor higher abundance of antibiotic resistance genes than conventionally produced?. Environ. Int..

[75] Sun Y., Qiu T., Gao M., Shi M., Zhang H., Wang X. (2019). Inorganic and organic fertilizers application enhanced antibiotic resistome in greenhouse soils growing vegetables. Ecotoxicol. Environ. Saf..

[76] Wei R., He T., Zhang S., Zhu L., Shang B., Li Z., Wang R. (2019). Occurrence of seventeen veterinary antibiotics and resistant bacterias in manure-fertilized vegetable farm soil in four provinces of China. Chemosphere.

[77] Peng S., Feng Y., Wang Y., Guo X., Chu H., Lin X. (2017). Prevalence of antibiotic resistance genes in soils after continually applied with different manure for 30 years. J. Hazard Mater..

[78] Adegoke A.A., Amoah I.D., Stenstrom T.A., Verbyla M.E., Mihelcic J.R. (2018). Epidemiological evidence and health risks associated xith agricultural reuse of partially treated and untreated wastewater: A review. Front. Public Health.

[79] Araujo S., Silva A.T., Tacao M., Patinha C., Alves A., Henriques I. (2017). Characterization of antibiotic resistant and pathogenic *Escherichia coli* in irrigation water and vegetables in household farms. Int. J. Food Microbiol..

[80] Makkaew P., Miller M., Fallowfield H.J., Cromar N.J. (2016). Microbial risk in wastewater irrigated lettuce: Comparing *Escherichia coli* contamination from an experimental site with a laboratory approach. Water Sci. Technol..

[81] Antwi-Agyei P., Cairncross S., Peasey A., Price V., Bruce J., Baker K., Moe C., Ampofo J., Armah G., Ensink J. (2015). A farm to fork risk assessment for the use of wastewater in agriculture in Accra, Ghana. PLoS ONE.

